# Complete sequence of six segments of fig mosaic virus from Iraq

**DOI:** 10.1128/mra.00763-23

**Published:** 2023-12-04

**Authors:** Shrooq Zagier, Osamah Nadhim Alisawi, Fadhal Al Fadhal

**Affiliations:** 1 Plant Protection Department, Faculty of Agriculture, University of Kufa, Najaf, Iraq; Katholieke Universiteit Leuven, Leuven, Belgium

**Keywords:** RNA-seq, bioinformatics, fig genome, fig mosaic virus

## Abstract

Here, we report the complete sequence of six segments of the Iraqi isolate of fig mosaic virus (FMV) named Kufa in fig plants. The sequence lengths of the six segments were determined. The phylogeny showed that four segments were closely related to Iranian isolates.

## ANNOUNCEMENT

**FIG 1:**
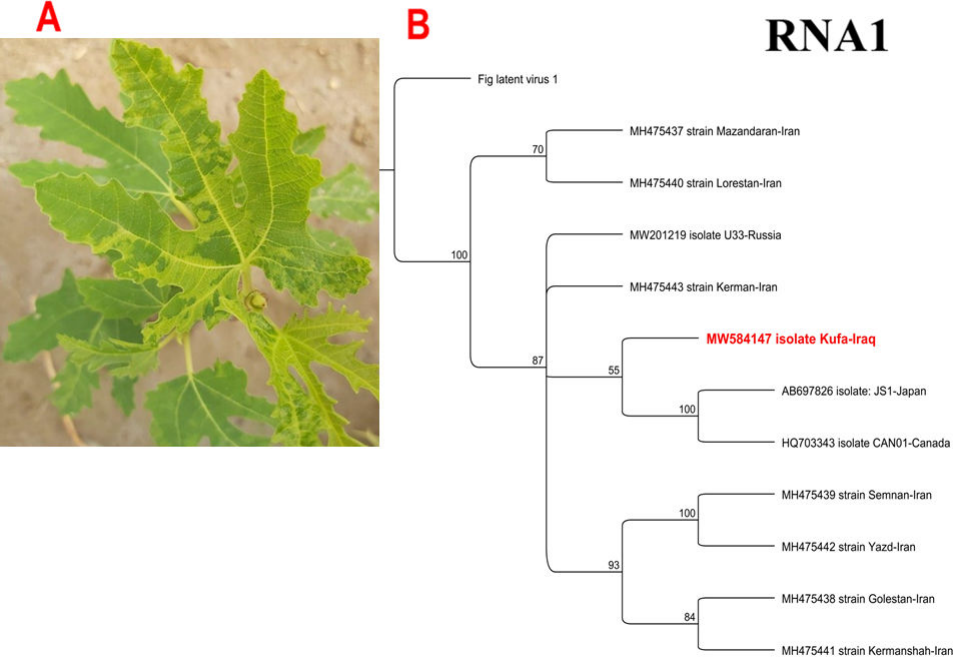
The leaves of fig show typical mosaic symptoms (**A**). The phylogenetic tree for fig mosaic virus segment RNA1 shows a close relationship to AB697826 and HQ703343 isolates from Japan and Canada, respectively. The trees were built by the Geneious tree builder, and the alignments of full-genome nucleotide sequences were performed with ClustalW. A maximum likelihood was constructed using the best substitution model Hasegawa-Kishino-Yano (HKY), and the tree was inferred with 500 bootstraps. The outgroup member was Fig latent virus 1 (**B**).

Fig mosaic virus (FMV) is a multipartite negative-sense RNA virus infecting fig trees worldwide that is efficiently transmitted by vegetative propagation and grafting but is not seed transmitted. It is also horizontally transmitted from plant to plant by the eriophyid mite *Aceria ficus* (1). FMV has been classified into the order *Bunyavirales* (-) sense ssRNA, the family *Fimoviridae*, and the genus *Emaravirus* (2). Virus particles are 720 nm long and 230 nm wide, with genome sequence lengths ranging from 1.212 to 7.09 kbp (3, 4). There are six RNAs in the FMV genome, including RNA1 that encodes RdRp (RNA-dependent RNA polymerase), RNA2 that encodes a glycoprotein, RNA3 that encodes a nucleocapsid protein (NP), RNA4 that encodes a motile protein, and RNA5 and RNA6 that encode proteins whose functions are still unknown (5). The virus has spread widely in fig-growing countries (6). Through PCR testing, most fig plant mosaic symptoms were confirmed to be caused by the fig mosaic virus in Iraq (7). Here, four symptomatic leaves showing mosaic symptoms from a single fig plant in Najaf Province were collected on 2 December 2020, and each leaf was cut into squares of 0.5 × 0.5 cm, and every square was immersed in a 5× volume of RNALater in a single Eppendorf tube and sent to DNA Link in the Republic of Korea (Fig. 1A). In accordance with the company’s instructions, total RNA was extracted using the RNeasy Plant Mini Kit (Qiagen, Hilden, Germany). To prepare the NGS library for RNA sequencing, the company used TruSeq Stranded Total RNA with the Ribo-Zero Plant library prep kit. RNA was subjected to whole genome sequencing (Platform: Illumina NovaSeq 6000; Application: WTS/mRNA), and the reads were trimmed using Trimmomatic v.0.39 and also BBduk v. 37.22 in Geneious Prime. Based on RNA-seq data, 39,933,818 clean and paired-end reads of 101 bases were generated. Clean and paired-end reads were mapped against the genomes of seven known fig-infecting viruses (Table 1) using the Geneious RNA mapper (sensitivity: medium-low sensitivity) in Geneious Prime 2023. We only identified reads mapping successfully against the six fig mosaic virus segments (8). Interestingly, the reads were aligned over the complete reference sequence, including the 5′- and 3′-UTR regions. All tools were run with default parameters unless otherwise specified. The result is consistent with what McGavin et al. (9) indicated that the FMV genome consists of six single RNA segments, including RNA1 and RNA4. Walia et al. (10) reported that each of the six RNA segments contains only one open reading frame, starting from 5′ to 3′, and each is wrapped separately in the virus capsid. A phylogenetic tree of the RNA1 segment showed a close relationship between the Kufa isolate and the AB697826 and HQ703343 isolates from Japan and Canada, respectively (Fig. 1B). The study revealed the first complete sequencing of the six segments and phylogenetic analysis of the fig mosaic virus in Iraq.

**TABLE 1:** Seven candidate fig virus genomes with accession numbers, total assembled reads, coverage depths, segment lengths, and GC content^*a*^

Viruses	Reference accession number		New genome accession number	Assembled reads	Coverage depths	Segment length (bp)	GC content %
Fig mosaic virus	NC_029562.1	RNA1 (RdRp gene)	MW584147.1	78,596	2,765	7,041	30.5
	NC_029565.1	RNA2 gene	MW584145.1	102,112	7,639	2,256	34.0
	NC_029563.1	RNA3 (N gene)	MW584142.1	179,542	31,746	1,496	32.3
	NC_029564.1	RNA4 gene	MW584143.1	363,852	50,500	1,469	35.5
	NC_029566.1	RNA5 gene	MW584146.1	16,328	4,171	1,743	30.7
	NC_029568.1	RNA6 gene	MW584144.1	19,672	4,384	1,217	33.3
Fig leaf mottle-associated virus 1	AM279676.1		0				
Fig leaf mottle-associated virus 2	FJ473383.1		0				
Fig mild mottle-associated virus	FJ611959.1		0				
Fig latent virus 1	FN377573.1		0				
Fig cryptic virus	NC_015494.1	Segment 1	0				
	NC_015495.1	Segment 2		0			
Fig fleck-associated virus	NC_015229.1			0			

^*a*^ The outcomes in the table resulted from mapping the reference genomes against the whole clean and paired-end RNA reads using the Geneious RNA mapper.

## Data Availability

This Whole Genome Shotgun project has been deposited in GenBank under accession no. SRR25146190. The version described in this paper is the first version. The complete sequence of FMV segments has been deposited in GenBank under accession numbers RNA1 (MW584147.1), RNA2 (MW584145.1), RNA3 (MW584142.1), RNA4 (MW584143.1), RNA5 (MW584146.1), and RNA6 (MW584144.1) and named as Kufa isolates.

## References

[1] Flock R , Wallace J . 1955. Transmission of fig mosaic by the eriophyid mite Aceria ficus. Phytopathology 45:52–54.

[2] Ishikawa K. , Maejima K , Komatsu K , Netsu O , Keima T , Shiraishi T , Okano Y , Hashimoto M , Yamaji Y , Namba S . 2013. Fig mosaic emaravirus p4 protein is involved in cell-to-cell movement. J Gen Virol 94:682–686. doi:10.1099/vir.0.047860-0 23152372

[3] Serrano L , Ramon J , Segarra J , Medina V , Achón MA , López M , Juárez M . 2004. New approach in the identification of the causal agent of fig mosaic disease. Acta Hortic 657:559–566. doi:10.17660/ActaHortic.2004.657.91

[4] Walia JJ , Salem NM , Falk BW . 2009. Partial sequence and survey analysis identify a multipartite, negative-sense RNA virus associated with fig mosaic. Plant Dis 93:4–10. doi:10.1094/PDIS-93-1-0004 30764262

[5] Ishikawa Kazuya , Maejima K , Komatsu K , Kitazawa Y , Hashimoto M , Takata D , Yamaji Y , Namba S . 2012. Identification and characterization of two novel genomic RNA segments of fig mosaic virus, RNA5 and RNA6. J Gen Virol 93:1612–1619. doi:10.1099/vir.0.042663-0 22513386

[6] Alhudaib K . 2011. Incidence of fig leaf mottle-associated virus and fig mosaic virus in Eastern province of Saudi Arabia. International J of Virology 8:128–132. doi:10.3923/ijv.2012.128.132

[7] Mohmmed RJ , AL Assie AH , Al Fahad MA . 2019. Molecular identification and biological resistance of the fig mosaic virus (FMV) on fig trees in Saladin governorate nurseries. Plant Archives (09725210) 19

[8] Kearse M , Moir R , Wilson A , Stones-Havas S , Cheung M , Sturrock S , Buxton S , Cooper A , Markowitz S , Duran C , Thierer T , Ashton B , Meintjes P , Drummond A . 2012. Geneious basic: an integrated and extendable desktop software platform for the organization and analysis of sequence data. Bioinformatics 28:1647–1649. doi:10.1093/bioinformatics/bts199 22543367 PMC3371832

[9] McGavin WJ , Mitchell C , Cock PJA , Wright KM , MacFarlane SA . 2012. Raspberry leaf blotch virus, a putative new member of the genus emaravirus, encodes a novel genomic RNA. J Gen Virol 93:430–437. doi:10.1099/vir.0.037937-0 22049090

[10] Walia JJ , Willemsen A , Elci E , Caglayan K , Falk BW , Rubio L . 2014. Genetic variation and possible mechanisms driving the evolution of worldwide fig mosaic virus isolates. Phytopathology 104:108–114. doi:10.1094/PHYTO-05-13-0145-R 24571394

